# SlimPLS: A Method for Feature Selection in Gene Expression-Based Disease Classification

**DOI:** 10.1371/journal.pone.0006416

**Published:** 2009-07-29

**Authors:** Michael Gutkin, Ron Shamir, Gideon Dror

**Affiliations:** 1 Blavatnik school of Computer Science, Tel Aviv University, Tel Aviv, Israel; 2 School of Computer Science, The Academic College of Tel-Aviv-Yaffo, Tel-Aviv, Israel; University of Manchester, United Kingdom

## Abstract

A major challenge in biomedical studies in recent years has been the classification of gene expression profiles into categories, such as cases and controls. This is done by first training a classifier by using a labeled training set containing labeled samples from the two populations, and then using that classifier to predict the labels of new samples. Such predictions have recently been shown to improve the diagnosis and treatment selection practices for several diseases. This procedure is complicated, however, by the high dimensionality if the data. While microarrays can measure the levels of thousands of genes per sample, case-control microarray studies usually involve no more than several dozen samples. Standard classifiers do not work well in these situations where the number of features (gene expression levels measured in these microarrays) far exceeds the number of samples. Selecting only the features that are most relevant for discriminating between the two categories can help construct better classifiers, in terms of both accuracy and efficiency. In this work we developed a novel method for multivariate feature selection based on the Partial Least Squares algorithm. We compared the method's variants with common feature selection techniques across a large number of real case-control datasets, using several classifiers. We demonstrate the advantages of the method and the preferable combinations of classifier and feature selection technique.

## Introduction

Classification of patient samples presented as gene expression profiles has become the subject of extensive study in biomedical research in recent years. One of the most common approaches is binary classification, which distinguishes between two types of samples: *positive*, or *case* samples (taken from individuals that carry some illness), and *negative*, or *control* samples (taken from healthy individuals). Supervised learning offers an effective means to differentiate positive from negative samples: a collection of samples with known type labels is used to train a classifier that is then used to classify new samples.

Microarrays allow simultaneous measurement of tens of thousands of gene expression levels per sample. Because typical microarray studies usually contain less than one hundred samples, the number of features (genes) in the data far exceeds the number of samples. This asymmetry of the data poses a serious challenge for standard learning algorithms–that can be overcome by selecting a subset of the features and using only them in the classification. This feature selection step offers several advantages:

- Improved performance of classification algorithms, thanks to removal of irrelevant features (noise).
- Improved generalization ability of the classifier, thanks to avoidance of over-fitting (learning a classifier that is too tailored to the training samples, but performs poorly on other samples).
- Fewer features, making classifiers more efficient in time and space.
- More focused analysis of the relationship between a modest number of genes and the disease in question.
- Less costly collection and storage of data.

Many feature selection techniques have been proposed. One of the most basic and popular methods involves *filters* [1], which select the subset of features as a pre-processing step, independent of the chosen classifier. Being computationally simple and fast, they can handle extremely large-scale datasets. Furthermore, feature selection needs to be performed only once, after which different classifiers can be evaluated [1]. Most filters are univariate, considering each feature independently of other features–a drawback that can be eliminated by multivariate techniques.

In this study we developed a novel feature selection technique based on the Partial Least Squares (PLS) algorithm [2]–[4], which we call SlimPLS. PLS aims to obtain a low dimensional approximation of a matrix that is ‘as close as possible’ to a given vector. SlimPLS is a multivariate feature selection method based on PLS that incorporates feature dependencies. We tested the performance of SlimPLS by five classifiers: linear Support Vector Machine (SVM), radial SVM, Random Forest, K-nearest-neighbors (KNN), and Naïve Bayes. 19 different case-control expression profile datasets comprising a total of 1547 samples were collected and used for training and testing. Our results show that the use of some SlimPLS variants leads to significantly better classification than that obtained with standard filters.

The use of PLS for classification is not new. In [5] the authors designed a procedure that entailed dimension reduction by PLS, followed by classification using the components constructed by PLS as the new extracted features; only a small subset of the total pool of genes was used for the construction of the components, selected by t-test. In [6] the authors extended this two-step procedure to support multiclass classification. Huang and Pan [7] used PLS and penalized regression for binary classification. First, *q* PLS components were constructed and a linear regression model was built using the components. Then, using a penalizing procedure, only genes with coefficients larger than some threshold *λ* were kept. Both *q* and *λ* were determined by cross validation. The classification itself is obtained by the penalized linear regression model. A similar procedure was employed in [8] in order to combine information from two different datasets of gene expression. Quite recently, Cao et al. [9] used PLS-SVD (a variant of PLS that uses singular value decomposition) together with Lasso Penalty in order to integrate data coming from different sources for classification. The combination of PLS and linear regression techniques was further studied in [10].

Fort and Lambert-Lacroix [11] described a classification using PLS with penalized logistic regression; like [5], this study ran the t-test filter before applying PLS. The discriminating abilities of PLS were studied in [12], where the connection between PLS and Linear Discriminant Analysis is shown. In addition, nonlinear extensions of PLS were also studied as kernel methods (e.g., [13], [14]), and their use together with SVM is described in [15].

All the above studies used PLS for classification, and when feature selection was involved, it was implicitly used. For example, in [7], where a penalizing process was applied to reduce the number of genes, the threshold parameter *λ*, which implicitly determines the number of features, was found using cross validation. Again, the goal in [7] was to construct a classifier rather than a feature selection technique *per se*.

The SlimPLS method is unique in that it focuses solely on feature selection; it does not propose a new classification procedure. As a result, it can be used as a pre-processing stage with different classifiers. Thus, we evaluate the performance of SlimPLS with different classifiers, and compare it to other feature selection methods and not to the PLS-based classification methods mentioned above.

## Methods

We first provide some background on PLS and then describe our algorithm.

### Partial Least Squares

Partial Least Squares (PLS) is one of a broad class of methods for modeling relations between sets of observed features by means of latent variables called components [16]. It is an iterative method that finds the relationship between a two-dimensional sample×feature matrix X and the class vector y of the samples; PLS was developed by Herman Wold and coworkers [2]–[4].

#### The basic algorithm

We shall use the following notation in the sequel. We denote a (column) vector by an underline 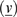, its j-th component by 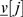, and its mean by the scalar 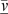. Matrices will be denoted by capital letters. An estimated or predicted parameter will be denoted by a tilde (e.g. 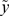). We use *n* for the number of samples (patients) and *k* for the number of required features (genes).

The basic goal of PLS is to obtain a low dimensional approximation of an *n*×*k* matrix *X* such that the approximation will be ‘as close as possible’ to a given *n*×1 vector 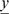. The simplest approximation is one dimensional: One seeks a *k*×1 vector 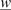 such that 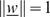 and 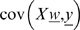 is maximal. 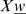 is called the *component* of *_X_* with respect to 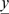, and is denoted by *t*. The *approximation error* is defined as 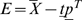 where 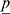 is a *k*×1 vector minimizing 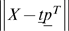. Similarly, the approximation error of 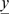 is defined as 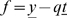, where *q* is a scalar minimizing 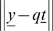. 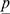 and *q* are called the *loadings* of *t* with respect to *X* and 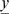, respectively.

The same process can be repeated iteratively by taking the approximation errors *E* and 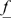 as the new *X* matrix and 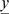 vector, and passing them to the next iteration. Hence, in the second iteration, a second component of *X* with respect to 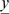 is computed; new approximation errors are obtained, which can subsequently be used to compute the third component, etc.

The substitution of *X* and 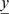 by their approximation errors is called *deflation*. This process can be repeated, and the desired number of components (hence, iterations) *a* is given to the algorithm as input.

This variant of PLS, which we shall use below, is sometimes called PLS1 [16], [17] to distinguish it from other variants that compute the approximations and the residuals in a slightly different fashion [16].

#### Classification with PLS

The use of PLS in classification is done in two parts–learning and prediction. In the learning part PLS extracts the 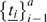 components by finding the weight vectors 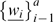 (recall that *a* is the number of components). These components are used to approximate the *X* matrix (expression matrix) and the 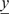 vector (class label vector).

In the prediction part the 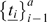 components are extracted from the query sample *z* using the weight vectors 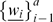 found in the learning phase. Together with the loadings 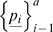 and 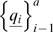 found earlier, PLS can then estimate the value of 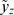, i.e., the estimated value of the class label of the query sample.

It should be emphasized that PLS is designed for regression, and as such does not predict the query sample's class. However, for a binary classification problem one can represent the class by as a numeric variable with two possible values, typically -1 and 1. In such a representation, PLS can output, for example, “0.92” as the query sample's approximated class label.

**Figure 1:**
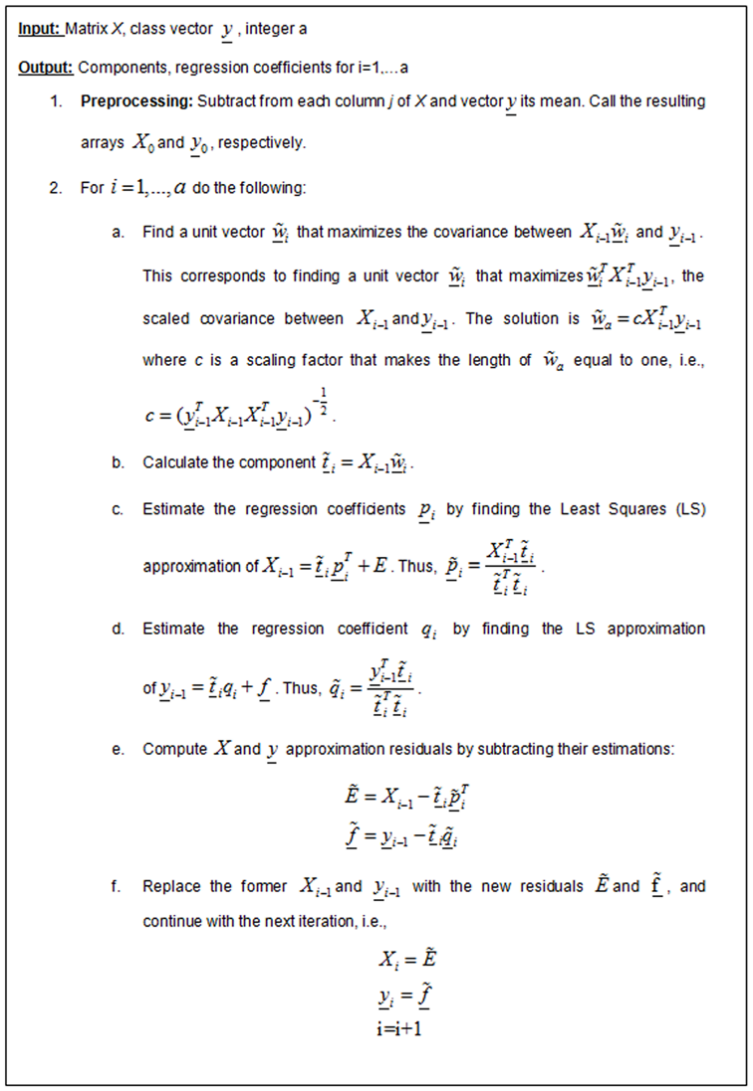
The learning stage of the PLS algorithm.

**Figure 2:**
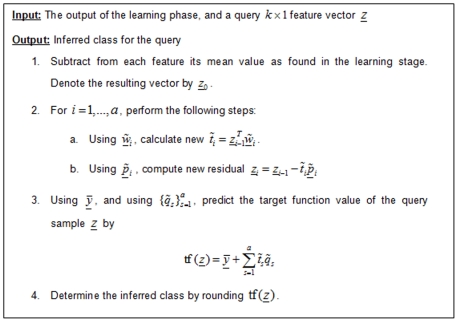
The prediction stage of the PLS algorithm.

The detailed learning algorithm is given in **Figure 1**; the prediction algorithm is given in **Figure 2**. See [17] for more details.

The complexity of each iteration of the learning stage (**Figure 1**) is 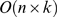, which is the complexity of calculations of matrix products needed for the component construction. Therefore, the total complexity of the learning stage is 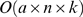.

The prediction stage (**Figure 2**; see [17] for more details) is similar to the learning stage: 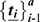 components are calculated using the 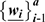 weight vectors found earlier. However, now we are dealing with only one sample (*z*), and not with a group of samples as in the learning stage. Therefore, each calculated component is actually a scalar. Because of that, in each iteration we have *O*(*k*) calculations, and the overall complexity of this step is 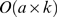.

### SlimPLS

Ranking-based filters usually utilize a univariate approach when selecting features. In some cases they can produce reasonable feature sets, especially if the features in the original set are uncorrelated. However, since the method ignores multivariate relationships, the chosen feature set will be suboptimal when the features of the original set are dependent. Some of the features will add little discriminatory power on top of previously selected features, although ranked relatively high individually [1], [18]. In these cases it is sometimes better to combine a highly predictive feature (one having a high rank according to some criterion) with some less predictive ones that correlate less with it. This way, the added features will be better able to ‘explain’ unexplained (or residual) ‘behavior’ of the samples than when only top-scoring features are used. Moreover, in some cases individual features are not highly predictive but gain predictive power when combined [19].

PLS is a good candidate for overcoming these problems. The PLS components are orthogonal and uncorrelated. Moreover, each component tries to approximate the residual (or error) left after using all former components. However, the method, in its original form, uses all the features without selection. Each component is constructed by a linear combination of all features using the weight vector *w*. By manipulating this vector, we can use PLS for feature selection or feature extraction, as described below. This way we choose only the most relevant features from each component before advancing to the next component. We call this technique SlimPLS.

#### Adapting PLS for feature selection

Several issues need to be addressed before applying PLS for feature selection:

1. How many features should be selected? The performance of classification and feature selection methods depends, among other factors, on the number of features that are selected. Too few features will have insufficient classification power, while too many features may add noise and cause over-fitting. Our analysis (see **Section 4.3.1**) showed clear improvement in performance when the number of related features was increased from 20 to 50, but no clear improvement when the number of features was increased beyond 50. We therefore used 20- and 50-feature configurations in our studies.
2. How many components of the PLS algorithm should be used? Typically, components computed at later iterations are much less predictive than former ones, as they approximate the residual, the residual of the residual, etc.;
3. When using several components, how many features should be selected from each component? Exactly how should they be selected?
4. Should one use the selected features themselves as the output of the process, or perhaps use the extracted PLS component (a linear combination of the selected original features) as the output?

We considered several possible answers to each question, and systematically tested algorithm variants implementing combinations of the choices.

#### The number of components and the number of features per component

We studied two possible approaches to partitioning the number of features across the PLS components.

- An equi-partition approach: Use n components and partition the features among them equally. The total number of features is assumed to be 50, unless specified otherwise. We call this type of variants **CONST**, and denote a specific variant by the parameter n. For example, CONST variant ‘5’ chooses a total of 50 features, 10 from each component, thus iterating over five components; variant ‘2’ uses two components, selecting 25 features from each one; ‘1’ uses one component.
- A dynamic partition approach based on computing p-values: This approach selects the number of components and the number of features from each component according to the properties of each component. A correlation coefficient is computed between each component and the original label vector 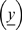, and a p-value for that correlation is calculated [20]. Components participate in the feature selection only if they achieve p-values lower than a given threshold *θ*. Then, the numbers of the features taken from each component are determined according to the distribution of the magnitudes of the p-values (−log(p-value)) of the relevant components. Specifically, the number of features *n_i_* selected from the *i*'th component is taken to be 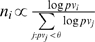 where 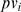 is the p-value associated with it.

For example, suppose the threshold *θ* is set to 5×10^−3^, and p-values for the correlation between the first ten components and the original label vector are calculated. The first component has a p-value of 1.7×10^−12^, the second one 5.2×10^−5^ and all other components have p-values larger than *θ*. Since only the first two components have p-values<*θ*, features will be selected using only these components. Now, we have to decide how many features will be selected from each component. Beginning with the pair of p-values (1.7×10^−12^, 5.2×10^−5^), we calculate their −log (p-value): (11.77, 4.28). The relevant proportions are therefore (0.73, 0.27). The number of features is selected from each component according to these proportions. For example, if we wish to select 50 genes, then 37 will be chosen from the first component and 13 from the second.

We call this variant **PVAL**, and denote it by the p-value used.

After selecting the desired number of features from a particular component, we modify the original weight vector 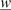 by putting zeroes in all entries other than the selected features and then re-normalizing 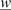. This way a modified component is constructed (using the modified 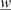 vector) instead of the original component. We compute approximations to the *X* matrix and the 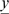 vector using this new component, and then continue to the next iteration, as in the original PLS algorithm.

#### Selecting features from a component

After determining the number of components and the number of features per component, the next step is to find the features themselves. We examined two possible approaches.

- Pick the top features in each component (variant **HIGH**): If we are to choose *k* features from a given component, we simply pick the *k* features that have the largest absolute weights in the vector 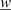 calculated for that component.
- A hill-climbing improvement approach (variant **HC**): We take the set of features obtained in (a) as a base set, and begin an improvement process using hill climbing [21], looking for a set of features of the same size that yields a lower approximation error ( ||E|| where 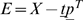, *t* is the component constructed using the selected set of features, and 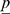 is its loading; see **Figure 1**). At each step of hill climbing, we randomly search for a better set of features, constructed by replacing one feature that currently belongs to the set by another feature that does not. The first switch that yields a lower error is chosen. This procedure terminates when no improvement is found after a given number of times (we used the number 50 in this study). The search is performed separately for each component.

#### Feature selection and feature extraction

Once we have determined the desired features in each component, we can use them in two ways:

- Use the selected features as the output. This approach is called **TOP**.
- Use the components as extracted features: In each component use the selected features to modify the original weight vector 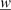 of that component, putting zeroes in all entries other than entries that belong to the selected features and then normalizing 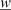. The constructed modified components are the output. Hence, these components are the new extracted features, and each of them is a linear combination of some original features. The total number of original features used is still as prescribed. In this approach the number of extracted features is the number of iterated PLS components. This approach is called **TCOMP**.

The full designation of the variants used will be a dash-separated tripartite name, where the first part is CONST/PVAL, the second HIGH/HC and the third TOP/TCOMP. The first part is a number: it denotes the number of components if it is a natural number, and the p-value otherwise. For example, 5×10^−2^-HC-TCOMP selects 50 features; the number of components and the number of features in each component are selected using the PVAL approach with a threshold of 5×10^−2^; features are optimized using hill climbing and components are used as the new extracted features.

**Table 1** summarizes the different SlimPLS variants described above. We categorize the variants into ‘families’, first depending on how many features they use from each component (constant, or using component p-values), and then - among those using p-values–depending on whether they take the highest scoring features or try to improve them via hill climbing.

**Table 1:** A summary of the SlimPLS variants and their properties.

Family	Feature Selector	Description
CONST	n-HIGH-TOP	Use n components, with an equal number of top features from each component
CONST	n-HIGH-TCOMP	As above, but use the modified components as the extracted features
CONST	n-HC-TOP	Use n components, with an equal number of features from each component; Select the features in each component by hill climbing from the K/n top ones, where K is the total number of features selected.
CONST	n-HC-TCOMP	As above, but use the modified components as the extracted features
PVAL-HIGH	p-HIGH-TOP	Select only components that show correlation p-value<p with the label vector; select the number of features from each component according to their relative p-values
PVAL-HIGH	p-HIGH-TCOMP	As above, but use the modified components as the extracted features
PVAL-HC	p-HC-TOP	Select only components that show correlation p-value<p with the label vector; select the number of features from each component according to their relative p-values; Improve by hill climbing
PVAL-HC	p-HC-TCOMP	As above, but use the modified components as the extracted features

In all variants the total number of features selected was set to 50 unless otherwise specified. n is a natural number and p is a real number 0<p<1. This difference in range of the first part of the name distinguishes the CONST from the PVAL variants.

## Results

### Datasets

We collected 19 datasets reported in the literature, with sample sizes ranging from 31 to 173 and containing between 2000 to 22283 features. The list of datasets appears in **Table 2**.

**Table 2:** The Datasets used in this study.

#	Dataset	Ref.	N	A	B	P
1	HD blood	[22]	31	14	17	22283
2	HD caudate	[23]	70	32	38	20223
3	Leukaemia	[24]	72	47	25	7129
4	HD cerebellum	[23]	66	27	39	20223
5	Prostate Cancer	[25]	102	50	52	12533
6	Breast Cancer	[26]	78	44	34	16783
7	Colon Cancer	[27]	62	40	22	2000
8	Crohn's Disease blood	[28]	101	42	59	22215
9	Breast Cancer	[29]	118	43	75	22215
10	Liver Cancer	[30]	60	20	40	7070
11	Breast/Colon Cancer	[31]	104	62	42	22215
12	Lung Cancer	[32]	86	62	24	7129
13	Liver Cancer	[33]	60	40	20	7129
14	Prostate Cancer	[34]	53	19	34	4344
15	Breast Cancer	[35]	58	28	30	2166
16	Breast Cancer	[36]	49	25	24	2166
17	Ovarian Cancer	[37]	54	30	24	22283
18	Neural tissue	[38]	150	100	50	12488
19	Myeloma and bone lesions	[39]	173	137	36	12625

Datasets 12–19 were used in [40]. For each dataset, N is the number of samples, A and B are the number of cases and controls, respectively, and P is the number of probes (features) measured. HD: Huntington's Disease.

Our goal was to find the more informative features. Because different features have different scales, the data had to be standardized before comparisons could be made. Each feature was linearly transformed to have a zero mean and variance 1. This data standardization is a common pre-processing step in microarray studies and was done previously when using PLS [7].

### Performance evaluation criteria

Using the benchmark of 19 datasets, we tested five classifiers and 36 feature selection algorithms–four filters (Pearson correlation coefficient [26], Welch test [20], which is a variant of T-test [41], Golub criterion [24] and mutual information [42]) and 14 SlimPLS variants (two CONST variants with one component: 1-HIGH/HC-TOP, eight PVAL variants: 5×10^−2^/5×10^−3^-HIGH/HC-TOP/TCOMP and four variants with two components: 2-HIGH/HC-TOP/TCOMP)–and selected a total of 20 and 50 features in each test. To avoid confusion, we will call a feature selection algorithm simply a *feature selector* (FS), and reserve the term *“combination”* for a combination of FS and classifier. Hence, we assessed a total of 180 combinations.

We used the R package [43] for the implementation of the SlimPLS methods, and used publicly available packages for the classifiers implementation: *e1701* [44] for SVM and Naïve Bayes, *class* [44], [45] for KNN, and *randomForest* [44], [46] for Random Forest. When running SVM, we used the grid 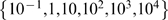 of possible values of C and found the best value of C using leave-one-out cross-validation on the training set. The Random Forest procedure was run with 1500 trees and 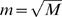 (where *M* is the total number of features and *m* is number of selected features that are used for branching on each node in each tree in the random forest; see [47] for more details). When running KNN, we used the grid {1,3,5,7} of possible values of *k* and found the best value of *k* using leave-one-out cross-validation on the training set. When running the *mutual information* filter, we used ten equal-sized bins. Ten bins showed better performance than fewer and more bins (results not shown).

A key question was how to evaluate performance. As some datasets are harder to classify than others, evaluating performance by the number of errors in each would give these datasets higher weight. Relative ranking of performance gives equal weight to all datasets, but ignores the absolute magnitude of the errors. For these reasons we used several criteria, each revealing a different aspect of the performance. Error rates were calculated using leave-one-out cross validation, and performance was measured according to two criteria:

- Rank sum p-value. Define a three-dimensional array *E* where 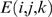 is the error rate of classifier *i* and feature selector *j* on dataset *k*. Hence, the dimensions of *E* are 5×36×19. Define an array *R* of the same dimensions, where 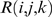 is the rank of 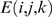 among 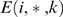. Hence, 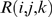 ranks feature selector *j* compared to all others for classifier *i* and dataset *k*. The score of a subset of feature selectors 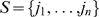 for classifier *i* is computed by comparing the distribution of the values 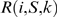 to the distribution of the values of 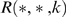, using the Wilcoxon rank-sum test [20]. This test determines the extent to which a particular group of values (e.g., the error rates of one feature selector) tends to have low rank compared to the rest. The p-values calculated on each dataset were combined using Fisher's method [48]. This score compares the different combinations of classifier and feature selectors, which means it also compares classifiers.
   Another comparison was made for each dataset. This time the distribution of the values 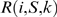 was compared to the distribution of the values of 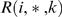 and a rank-sum score was computed as above. This score was used to compare the feature selectors using different individual classifiers, since it evaluates the performance of the different feature selectors using a particular classifier.
   We used the two scores defined here to compare combinations of a family of feature selectors (or a subset of a family) and a classifier. In other words, we did not compare one feature selector to another, but compared groups of similar variants.
- Binomial tail p-value. We used only 50 features with this method. Let 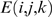 be defined as before, using only the 50 features version of the feature selectors. Hence, the dimensions of *_E_* are 5×18×19. 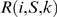 is defined as the rank of 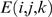 among 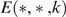. To compare two combinations 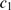 and 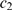, where the first one uses classifier 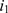 and FS 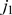, and the second one uses classifier 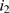 and FS 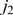, we compare the two vectors 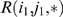 and 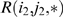. Let 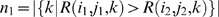 and let 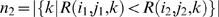. Then 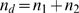 is the number of datasets in which the ranks using combination 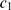 and combination 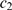 differ. Our null hypothesis is that the two combinations show similar performance. In other words, after removing the entries that have identical values we assume that 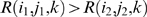 has a probability of 0.5. Therefore, 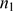 has a binomial distribution 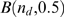. The p-value for observing at least 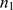 cases where combination 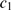 is ranked above combination 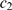 is: 
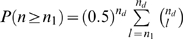
.

### The effect of the number of features

We first tested how performance changed when different numbers of features were selected. For a particular feature selector *j* using a particular classifier *i*, we calculated the average error rate achieved by this combination. Formally, we calculated 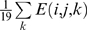. Notice that we use here the error rates, since we are evaluating the performance of a particular feature selector using different numbers of features.

**Figure 3:**
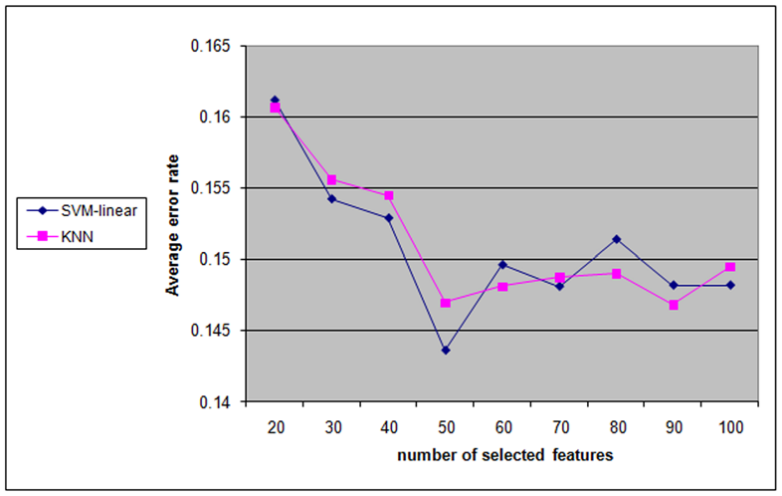
Performance as a function of the number of selected features. Performance is measured by the average error rate of six different SlimPLS-based feature selectors using the KNN and SVM-radial classifiers, for different numbers of selected features.

For a given classifier, we calculated this average error rate for six different variants of SlimPLS: 1-HC/HIGH -TOP, 5×10^−2^/5×10^−3^-HC-TOP and 5×10^−2^/5×10^−3^-HC-TCOMP (see **Table 1** for variant definitions) using nine different numbers of selected features: 20, 30, … 100. Then, the average error rate over these feature selectors was calculated for each number of selected features. The results for the classifiers SVM-radial and KNN are summarized in **Figure 3**.

An improvement in performance is evident when the number of selected features increased from 20 to 50. No significant improvement is noticeable when the number increased further. Therefore, the following focuses only on the 50-feature configurations, unless otherwise stated.

### The effect of the classifier

The average rank-sum p-values of each classifier were calculated over three groups of feature selectors:

- Filters: the four filters used in this work.
- CONST: the two TOP variants that choose all features from one component, 1-HIGH/HC-TOP.(We excluded the TCOMP variants since they report only one extracted feature, and some of the classifiers that we tested do not support classification with one feature).
- PVAL-HC: the four variants that choose a variable number of features per component, depending on the p-values: 5×10^−2^/5×10^−3^-HC-TOP/TCOMP.

**Figure 4:**
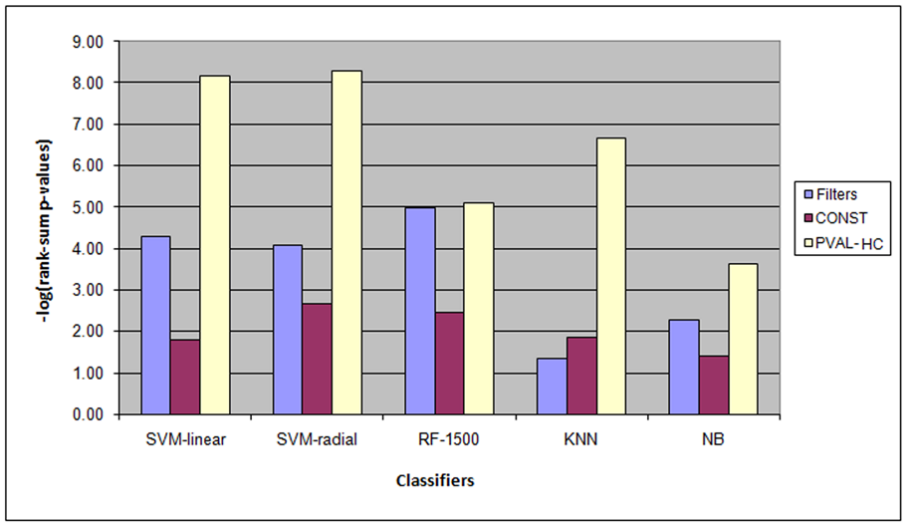
Performance of different classifiers using three groups of feature selectors. −log(p values) of the combined Wilcoxon rank-sum tests for three groups of methods using five different classifiers are shown. See text for the group definitions.

The results are summarized in **Figure 4**. For the PVAL-HC variants, SVM (linear and radial) and KNN showed better performance than RF and NB. Moreover, these three classifiers together with the four PVAL-HC variants achieved the highest scores among all combinations. When using filters, the RF classifier performed best and SVM classifiers second. The filters performed worst with the KNN classifier. The difference in the performance of PVAL-HC variants and filters was the most pronounced with this classifier (see *Discussion*).

**Figure 4** shows that SVM-linear and SVM-radial classifiers produced similar results. When the NB and RF classifiers were used, the PVAL-HC FS variants outperformed other feature selectors, but less dramatically than with the other classifiers.

**Figure 5:**
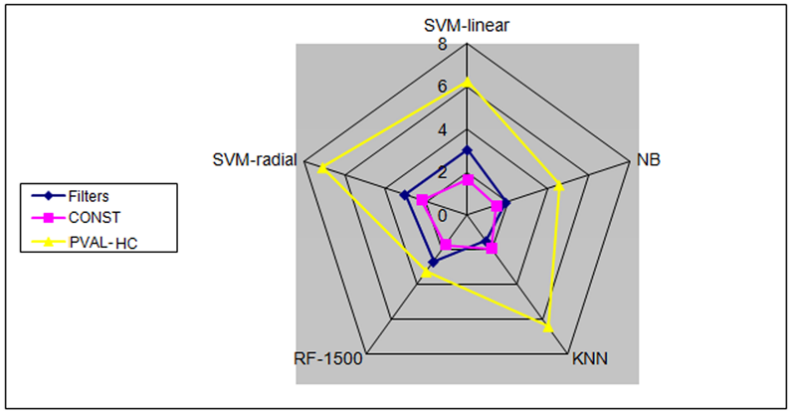
Performance of three groups of feature selectors calculated separately for each classifier, according to the classifier-specific rank-sum p-value. −log(p-value) of the combined Wilcoxon rank-sum tests for the three groups using five different classifiers are shown. The concentric pentagons show the −log(p-value) scale. The results on separate classifiers are shown on the separate axes. This representation is intended to emphasize the relative performance of each FS on each classifier separately, and does not present relative performance across classifiers. See text for the description of the families.

To get a clearer understanding of the influence of the feature selectors on the different classifiers, we performed the second variant of the rank-sum test (see ***Results*** **,** ***Performance evaluation criteria a*** **, and** **Figure 5**). This time the comparison between the different feature selectors for each specific classifier was done separately. The PVAL-HC variants showed a clear advantage over the other feature selectors. The differences were minor with RF, but stronger with the other classifiers.

As in the previous test (**Figure 4**), the greatest advantage of PVAL-HC feature selectors over the filters was attained when the KNN classifier was used.

### The effect of the feature selectors

**Figure 6:**
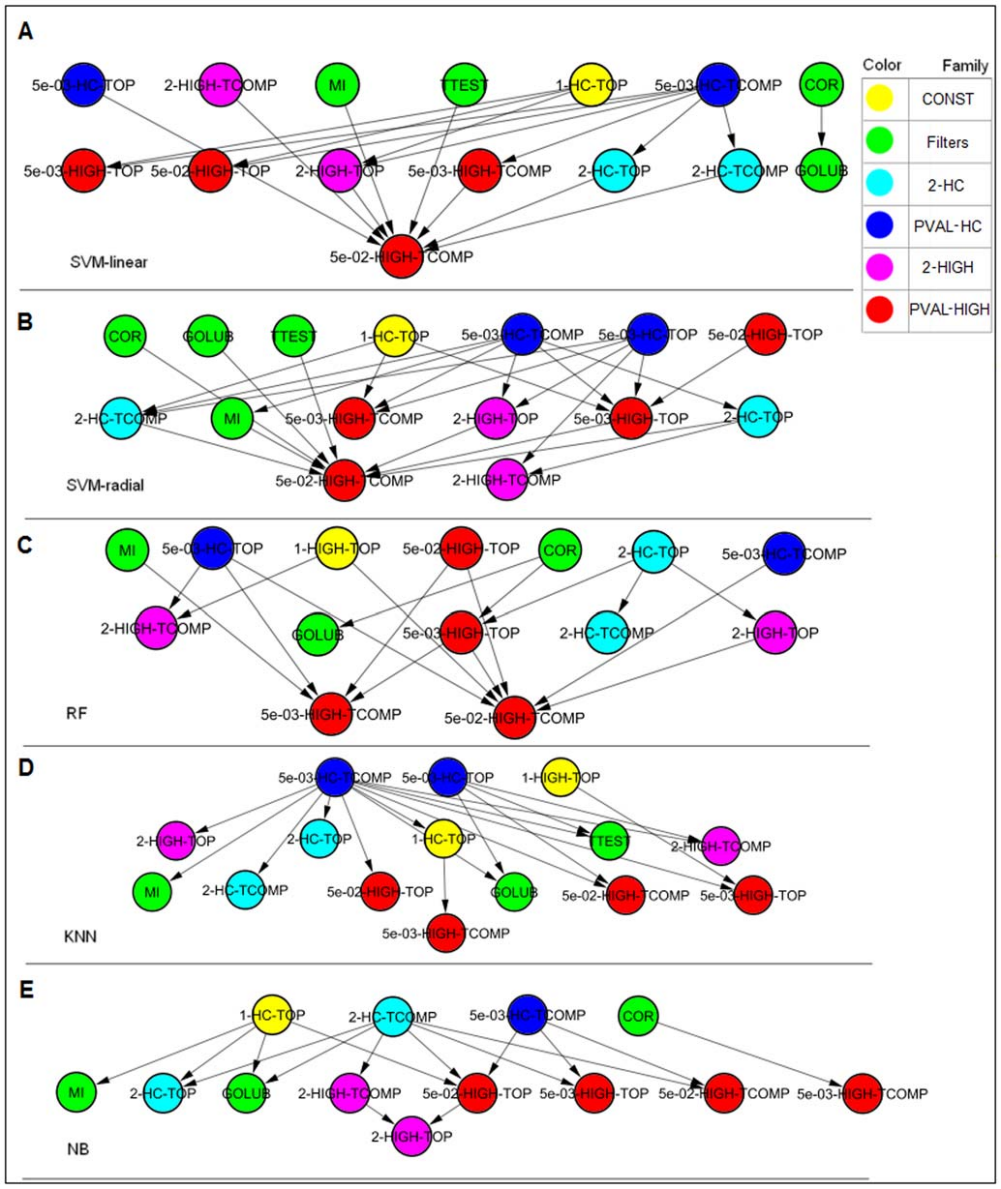
Dominance maps for comparing feature selectors. The comparison is done separately for each classifier: (A) SVM-linear (B) SVM-radial (C) Random Forest (D) KNN (E) Naïve Bayes. An edge X→Y indicates that X significantly outperformed Y. In (D) the second and the third layers from the top were originally one layer that was divided into two rows for display purposes only. Methods in upper layers performed better than methods in lower ones.

**Figure 6** summarizes the comparisons between different feature selectors using *dominance maps*. These are directed graphs in which each node is a combination and a directed edge from 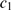 to 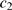 indicates that combination 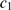 performed significantly better 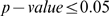 than combination 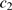. Performance is measured using the binomial tail for the relative accuracy of the two combinations across the datasets. See ***Results*** ***(Performance evaluation criteria b)*** for more details. We constructed five maps, one for each classifier. Singletons–combinations that were not significantly comparable to any other combination (corresponding to isolated vertices in the map)–were omitted. Transitive edges were also removed: if there were three edges A→C, A→B and B→C, edge A→C was removed. Out of the four variants of the PVAL-HC group, only two were taken: 5×10^−3^-HC-TOP/TCOMP.

One can see a clear tendency of the PVAL-HC variants (the blue nodes) to appear in the upper row, the location of the better performing FS for the given classifier. The PVAL-HC nodes also tended to have more outgoing edges, showing dominance over many other feature selectors. A very strong dominance of the PVAL-HC variants was observed with the KNN classifier (**Figure 6(d)**). In addition to the PVAL-HC variants, the Filters (green nodes) and the CONST-50-50 variants (yellow nodes) also tended to perform well.

Four feature selectors were never dominated by others: 1-HIGH-TOP, 5×10^−3^-HC-TOP, 5×10^−3^-HC-TCOMP and COR, the correlation filter (combinations that were singletons are not shown on the maps).

### Evaluation of the leading combinations

**Figure 7:**
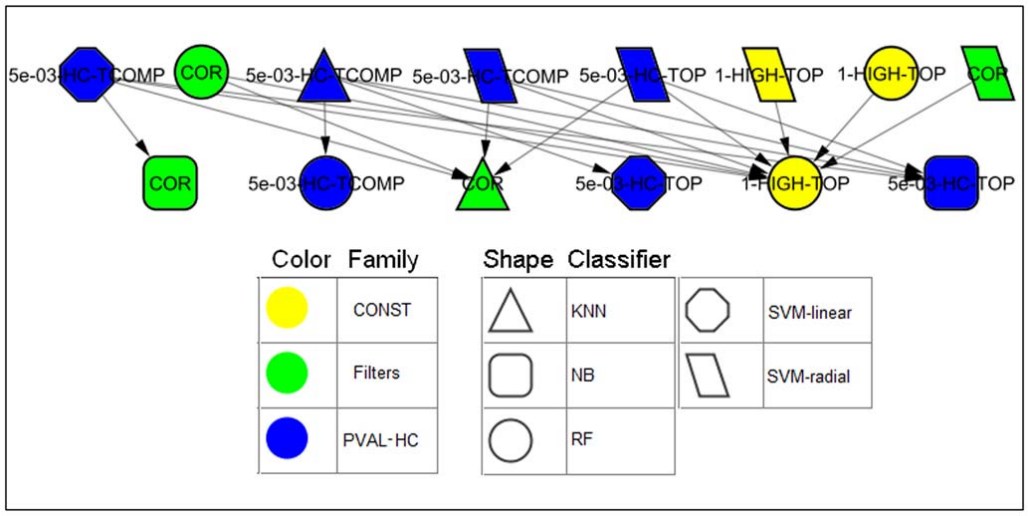
A dominance map comparing the performance of four leading feature selectors using all classifiers. Additional singletons not shown in the picture: KNN - 5×10^−3^-HC-TOP and 1-HIGH-TOP; SVM-linear–1-HIGH-TOP and COR; RF - 5×10^−3^-HC-TOP; NB - 5×10^−3^-HC-TCOMP.

The above comparison evaluated feature selectors for each classifier separately. Because comparisons of all combinations of feature selector and classifier yield complex, hard-to-interpret results (see [49]), we calculated another dominance map (**Figure 7**) containing only the four leading feature selectors and all the classifiers. We chose only those feature selectors that were not dominated by any of the others in the previous analysis: 1-HIGH-TOP, 5×10^−3^-HC-TOP, 5×10^−3^-HC-TCOMP and COR.

All four combinations using SVM-radial were undominated. The combinations of SVM-linear and KNN with 5×10^−3^-HC-TCOMP dominated the largest number of others.

All combinations involving the Naïve Bayes classifier with the exception of the combination with 5×10^−3^-HC-TCOMP were dominated by others. This is consistent with the classifiers comparison (**Figure 4**). The generally poorer performance of Naïve Bayes compared to the other classifiers is in line with the prevailing view that discriminative classifiers are almost always preferred (see e.g. [50])

### Robustness

A key question in feature selection is robustness: how stable is the set of selected features when the data are perturbed? To address this question, we found the features for each dataset that were selected at least half the time in the leave-one-out cross-validation iterations, and then averaged this number over all datasets.

As expected, the filters achieved high robustness. For example, when a total of 50 features were selected, the t-test filter found an average of 48 features that were selected in at least half of the iterations. The HIGH variants achieved an average of 43–46 features, except for the 1-HIGH-TOP variant, which achieved an average of 49.1 features. The 5×10^−3^-HC-TOP variant achieved an average number of 36.7 features and the 1-HC-TOP an average of 40.8 features (the matching TCOMP variants are not mentioned as they use the same mechanism for feature selection and therefore would have the same scores).

The drop in the average for the HC variants is due to the hill climbing procedure. As hill climbing randomly tests candidate replacement features, it can find, say, two distinct sets that perform similarly as a group. Thus, HC variants may consistently find the ‘core’ set of features, but may find different subsets of features to provide the full ‘explanation’ of the domain.

More details on the robustness comparison can be found in [49].

### Running Times

The running time of a feature selector can be divided into two components: feature selection time and classification time. The running times for each component are summarized in **Table 3**. The HIGH variants exhibited comparable feature selection time to the filter, and had a faster feature selection time when only one PLS component was used. The HC variants showed slower feature selection times than the filters, due to the hill climbing search, which takes extra time after the initial set of features is found. With regard to the classification time, the TOP variants showed times similar to those of the filters, while the TCOMP variants were faster, having to test fewer (extracted) features than the TOP variants or the filters.

**Table 3:** Average running time of different feature selection techniques.

Feature selection technique	Feature selection time	Classification time
Correlation filter	4.00	4.86
1-HC- TOP	9.71	4.71
5×10^−2^-HC-TCOMP	27.00	3.00
5×10^−2^-HC-TOP	26.29	5.43
5×10^−3^-HC-TCOMP	27.57	2.43
5×10^−3^-HC-TO^P^	27.86	4.43
1-HIGH-TOP	1.43	4.29
5×10^−2^-HIGH-TCOMP	4.86	1.71
5×10^−2^-HIGH-TOP	4.86	4.43
5×10^−3^-HIGH-TCOMP	4.57	1.71
5×10^−3^-HIGH-TOP	4.43	4.71

Times are in seconds per iteration of the leave-one-out cross validation. The SVM-radial and KNN classifiers were used after the feature selection phase. These tests were done using a Linux platform with Intel Xeon 5160 CPU, 3.00 GHz clock, and 4 GB of RAM.

## Discussion

We have described a new algorithm for feature selection based on the Partial Least Squares method, and used a variety of classification algorithms to compare it to filter methods. Our tests on real case-control biological datasets show an advantage of the new method over filter methods.

We focus here on the 50-feature configuration since in most cases, classifiers achieved a lower error rate with that configuration compared to the 20 selected features configuration, and, increasing the number of features beyond 50 brought no consistent improvement (**Figure 3**, see also [49]).

The PVAL-HC variants of SlimPLS tended to outperform the other tested variants (**Figure 5** and **Figure 6**). These variants select the number of features per component based on their significance and try to improve the feature set by local search. Among these variants, the TCOMP variants, which employ feature extraction (but still use 50 features only), tend to achieve slightly better results than the TOP variants. This is not surprising since the components, which are actually the extracted features, are found in a way that maximizes the match to the class vector; that is, the components are chosen to provide a good approximation of the class prediction. The TOP variants use the selected features for classification, but without the formulas that dictate how to re-build these components (the weight vectors). This leaves the task of constructing the formulas–of finding the relevant relationships between these features in order to get a good classification–to the classifier. When the TCOMP variants are used, we usually get one to four components that already incorporate some ‘collective’ behavior of features found by SlimPLS. Moreover, each component approximates the residual or the ‘unexplained’ behavior of the previous component. Thus, these new extracted features contribute more information to the classification.

The better performance of the PVAL-HC variants compared to the filters is more dramatic when the KNN classifier is used (**Figure 5**). This is an interesting result in view of the high sensitivity of the KNN classifier to the selected features [51]. It appears to indicate the ability of SlimPLS feature selection techniques to find good informative groups, especially when these groups are translated into new features, extracted in TCOMP variants. The combination of the KNN classifier and the 5×10^−3^-HC-TCOMP feature selector had the lowest average error-rate. The combination of KNN and 5×10^−2^-HC-TCOMP had the second lowest average error-rate (see [49]). SVM-radial also showed consistently high performance when the better feature selectors were used (see **Figure 7**).

As mentioned above, HC variants may consistently find the ‘core’ set of features, but they may also find different subsets of features to fully ‘explain’ the domain. Further research on the ‘core’ set is needed since, despite the random process, these features are repeatedly selected.

The SlimPLS method appears promising. Future work should examine automatic calculation of the p-value threshold in the PVAL variants (e.g., the faster the significance drops for constructed components, the more significant a component will have to be to pass the threshold), and using a minimum number of features per component for better ‘capture’ of the component's behavior. Another possibility is to stop the local search after a prescribed number of runs, or after attaining a desired percentage of improvement of the objective function, and allow one of the currently selected features to be replaced by another one that is not, only if the improvement (absolute or relative) of the target function is higher that some threshold. Different local search algorithms such as simulated annealing could be applied. It would also be interesting to use biology-based logic in the local search. The greedy search tries to find a switch that improves the target function. A mechanism can be inserted that prevents some switches, even if they improve the target function. For instance, one gene can be switched with another only if the two belong to the same pathway in a given biological network. Or, a switch can be allowed only if there are representative genes from at least (or at most) *k* different modules of the biological network in the resulting subgroup.
